# Bioadhesive eutectogels supporting drug nanocrystals for long-acting delivery to mucosal tissues

**DOI:** 10.1016/j.mtbio.2022.100471

**Published:** 2022-10-25

**Authors:** María Beatrice Bianchi, Chunyang Zhang, Elise Catlin, Giuseppina Sandri, Marcelo Calderón, Eneko Larrañeta, Ryan F. Donnelly, Matías L. Picchio, Alejandro J. Paredes

**Affiliations:** a Department of Drug Sciences, University of Pavia, Viale Taramelli 12, 27100, Pavia, Italy; b School of Pharmacy, Queen's University Belfast, Medical Biology Centre, 97 Lisburn Road, Belfast, BT9 7BL, UK; c POLYMAT, Applied Chemistry Department, Faculty of Chemistry, University of the Basque Country UPV/EHU, Paseo Manuel de Lardizabal 3, 20018, Donostia-San Sebastián, Spain; d IKERBASQUE, Basque Foundation for Science, 48009, Bilbao, Spain

**Keywords:** Nanocrystals, Eutectogels, Bioadhesion, Drug delivery, 3D printing, Curcumin

## Abstract

Eutectogels (Egels) are an emerging class of soft ionic materials outperforming traditional temperature-intolerant hydrogels and costly ionogels. Due to their excellent elasticity, non-volatile nature, and adhesion properties, Egels are attracting a great deal of interest in the biomedical space. Herein, we report the first example of adhesive Egels loading drug nanocrystals (Egel-NCs) for controlled delivery to mucosal tissues. These soft materials were prepared using gelatin, glycerine, a deep eutectic solvent (DES) based on choline hydrochloride and glycerol, and nanocrystallised curcumin, a model drug with potent antimicrobial and anti-inflammatory activities. We first explored the impact of the biopolymer concentration on the viscoelastic and mechanical properties of the networks. Thanks to the dynamic interactions between gelatin and the DES, the Egel showed excellent stretchability and elasticity (up to ≈160%), reversible gel-sol phase transition at mild temperature (≈50 ​°C), 3D-printing ability, and good adhesion to mucin protein (stickiness ≈40 ​kPa). *In vitro* release profiles demonstrated the ability of the NCs-based Egel to deliver curcumin for up to four weeks and deposit significantly higher drug amounts in excised porcine mucosa compared to the control cohort. All in all, this study opens new prospects in designing soft adhesive materials for long-acting drug delivery and paves the way to explore novel eutectic systems with multiple therapeutic applications.

## Graphical abstract

**Figure:**
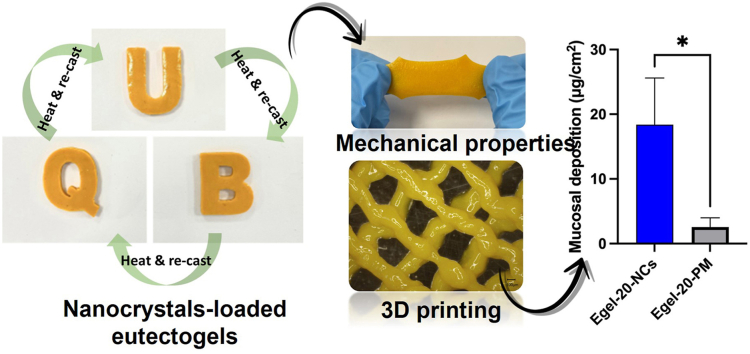

## 1 Introduction

Deep eutectic solvents (DES) are an attractive class of low-cost electrolytes featured by an abnormal depression in their eutectic point temperature compared to that of an ideal liquid mixture [1,2]. The immobilization of these intriguing mixtures in polymer matrixes offers new opportunities for generating functional gel materials with promising applications in healthcare [3,4]. Due to the unique ionic conductivity of the DES component, recent studies have mainly focused on exploring eutectogels as wearable sensors or bioelectrodes, aiming to replace expensive and non-biocompatible ionic liquid gels [[5], [6], [7]]. However, eutectogels also benefit from several features that can be exploited in controlled drug delivery systems, such as good biocompatibility, adhesiveness, stiffness matching biological tissues, and non-volatile nature [8]. Furthermore, DES have proved to dramatically increase the solubility, stability, and permeability of drugs, becoming versatile delivery systems for nasal, transdermal, and oral administration with improved bioavailability [[9], [10], [11]].

Drug nanocrystals (NCs) are sub-micrometric particles (<1 ​μm) composed of 100% drug in a crystalline state, featured by having increased saturation solubility, dissolution rate, and bio-adhesiveness, allowing to boost the biopharmaceutical performance of hydrophobic drugs [[12], [13], [14]]. The formulation of NCs has become one of the most preferred techniques for overcoming poor drug solubility, both in industrial and academic laboratories, allowing for the development and approval of several NCs-based medicines suitable for multiple administration routes [12,15]. Therefore, combining DES and NCs could result in advantageous drug delivery platforms with outstanding features, avoiding the rapid clearance of NCs from the application site. Consequently, these technologies hold substantial potential for long-acting drug delivery, which is especially appealing in mucosal tissues, where multiple challenges related to mucosae physiology have to be overcome [16]. These include a limited area, secretion of fluids, enzymatic degradation, poor tissue penetration, and palatability in the case of oral products [17]. However, scarce studies on eutectogels as drug delivery systems have been conducted so far, and to the best of our knowledge, NCs formulations have not been considered in the formulation design [18]. Poly(2-hydroxyethyl methacrylate) eutectogels hosting choline chloride (ChCl)/ascorbic acid (2:1) or ChCl/fructose (2:1) DES have been recently developed for oral delivery of sunitinib malate or indomethacin, respectively [19,20]. Furthermore, some hydrogel systems incorporating DES as drug solubilizes or bioactive liquids have been reported [21,22]. Freire et al. prepared alginate hydrogels by adding glycerol (Gly)/arginine (4: 1) DES/water mixture as a solubility enhancer of ibuprofen [23]. In another recent study, amino acids/citric acid (3:1) DES were integrated into Carbomer® 940 hydrogels for skin delivery of “Sanwujiaowan” extract, a Chinese herb medicine [24]. In this context, there is still a profound lack of studies addressing the design of functional eutectogels for pharmaceutical and delivery technologies.

**Fig. 1:**
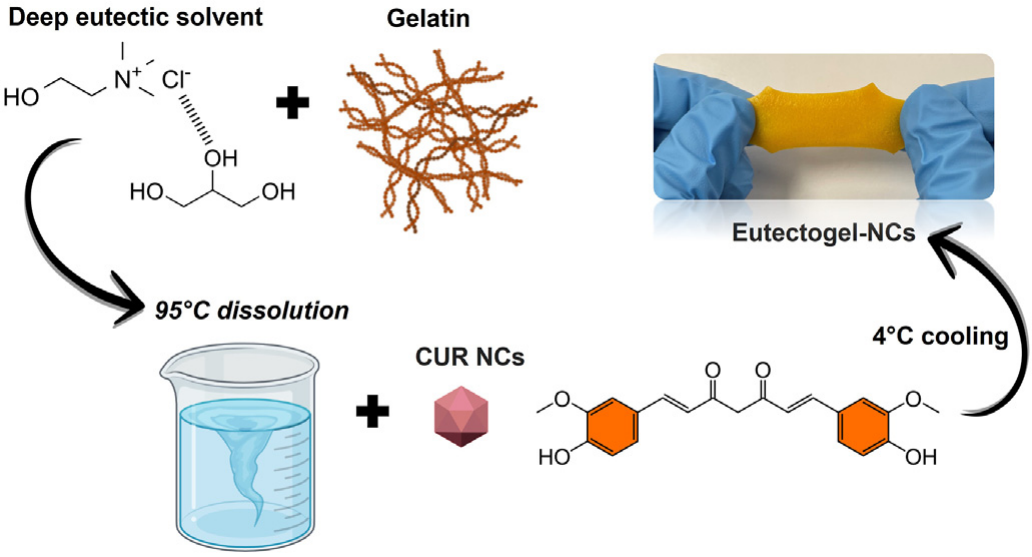
Schematic illustration of the preparation of gelatin-based eutectogels integrating CUR-NCs.

Herein, we propose the synergistic combination of drug NCs and a eutectic mixture supported into a gelatin matrix to obtain stretchable and adhesive eutectogels for long-acting mucosal delivery (Fig. 1). Curcumin (CUR) was chosen as a model hydrophobic drug to prepare NCs and glycerine, ChCl/Gly (1:3), as the eutectic system. This DES has already demonstrated a powerful solubility enhancement of CUR, providing a great option to maximize its therapeutic outcome [25]. Firstly, we focused on the effect of the gelatin concentration on the eutectogels' viscoelastic and mechanical properties. The most promising formulation was loaded with CUR-NCs, and the resulting eutectogels nanocomposites were analyzed in terms of their physicochemical properties, 3D-printing ability, and capability for drug delivery in an *ex vivo* porcine mucosa model.

## 2 Materials and methods

### 2.1 Chemicals

Curcumin (CUR), synthetic, purity >97.0% was purchased from Tokyo Chemical Industries (London, UK). Gelatin from bovine skin gel strength ∼225 ​g bloom, type B, choline chloride ≥99% (ChCl), and glycerol ≥99% (Gly) were obtained from Sigma Aldrich Co (Gillingham, UK). Poloxamer 188 (P188) was obtained from BASF Chemical Company (Ludwigshafen, Germany). Zirconia beads partially stabilized with Yttria (type YTZP) with a diameter of 0.1–0.2 ​mm were obtained from Chemco (Guangfu, China). Ultra-pure water was used in all the experiments (Elga PURELAB DV 25, Veolia Water Systems, Dublin, Ireland). All other reagents were of analytical grade and purchased from standard commercial suppliers.

### 2.2 Preparation of eutectogels

The DES was prepared by dissolving ChCl and Gly (1:3 ​M ratio) at 95 ​°C under stirring. Once the DES was formed, different amounts of gelatin were added to the system. Drug-free blank eutectogels containing 10, 15, 20, and 25% w/v of gelatin were prepared, with their formulation codes being Egel-10, Egel-15, Egel-20, and Egel-25, respectively. After the gelatin was fully dissolved, the resultant solution was poured into silicone moulds and stored overnight at 4 ​°C to allow the gel formation. For preparing the drug-loaded eutectogels (Egel-NCs), 50 ​mg of the solid NCs were added per gram of the molten gel (final drug concentration: ≈5 ​wt%) and immediately blended without heating by dual asymmetric centrifugation in a SpeedMixer™ (DAC 150 FVZ, High Wycombe, England) for 20 ​s at 3500 ​rpm. The resultant blend was poured into silicone moulds and allowed to cool at 4 ​°C overnight. The obtained gels were stored in the fridge and protected from light until used for characterization.

### 2.3 Fourier transform infrared spectroscopy (FTIR)

Infrared spectroscopic patterns of CUR, CUR-NCs, and Egel-NCs were obtained using an Accutrac FTIR (FT/IR-4100 Series, Jasco, Essex, UK) equipped with Diamond MIRacle™ A.T.R. The region studied covered the range of 4000–600 ​cm^−1^ with the resolution set at 4.0 ​cm^−1^. Each spectrum was obtained using an average of 64 repeat scans.

### 2.4 Rheological measurements

Amplitude, frequency, and temperature sweep experiments were performed in a stress-controlled Anton Paar Physica MCR101 rheometer, employing 8 and 25 ​mm parallel-plate geometries. Amplitude sweep experiments were carried out from 0.1 to 100% strain at a constant frequency of 1 ​Hz at 25 ​°C. Frequency experiments were performed from 0.1 to 100 ​rad/s at 1% strain at 25 ​°C. Temperature sweep experiments were carried out from 0 to 80 ​°C at 4 ​°C/min and a fixed frequency of 1 ​Hz and 1% strain (in the linear range of viscoelasticity of the materials).

### 2.5 Tensile test

For the tensile tests, gel specimens with bone shapes of 25 ​mm in length and cross-section 3.5 mm ​× ​1 ​mm were cut. Tests were carried out using a TA HD plus Texture Analyzer equipment (Texture Technologies) at 23 ​°C, 50% relative humidity, and an elongation rate of 25 ​mm/min. At least five specimens of each sample were tested.

### 2.6 Manufacture of CUR nanocrystals

CUR NCs were prepared by a previously reported slightly adapted lab-scale media milling technique [26]. Briefly, 100 ​mg of CUR were placed in a 10 ​mL glass vial together with 5 ​mL of 0.5, 1, 1.5, or 2% w/v P188 solutions, 4.5 ​mL of zirconia beads (Chemco beads, Suzhou, China) and 2 magnetic bars (25 ​× ​8 ​mm), which were vertically aligned. The system was agitated using an RCT BASIC magnetic stirrer (IKA, Staufen, Germany) at 1200 ​rpm. After grinding for 24 ​h, the CUR nanosuspensions (NSs) were separated from the milling media using a 200-mesh sieve. The resultant NSs were dried for 26 ​h in a freeze-dryer to collect the NCs, which were gently ground to a fine powder using mortar and a pestle.

### 2.7 Particle size determination

The particle size of the pristine drug was determined by laser diffraction using a Mastersizer® 3000 equipped with a Hydro® cell (Malvern Panalytical Ltd, Worcestershire, England). For this purpose, a 2 ​mg/mL suspension of the drug was prepared using P188 2% w/v as the dispersant. The sample was further diluted until the scattered light intensity was between 5 and 15% and analyzed six times. The results were expressed in terms of the De Brouckere mean diameter ([D4,3]) and volume density distribution. The particle size, polydispersity index (PDI), and zeta potential of the NCs were determined using a NanoBrook Omni® analyzer (Brookhaven, New York, USA). To this end, 5 ​μL of the NS were dispersed manually in 4 ​mL of water, transferred to disposable plastic cells, and analyzed. The equilibration time was set at 3 ​min, and determinations were made at 25 ​°C. Results were expressed as mean values ​± ​standard deviation (mean ​± ​SD, *n* ​= ​3).

### 2.8 Preparation of drug/P188 physical mixture

A drug plus P188 physical mixture (PM) was prepared and used as a control throughout the work. The PM containing 100 ​mg of CUR and 75 ​mg of P188 was prepared using the SpeedMixer™ for 5 ​min at 3500 ​rpm. This composition was equivalent to the dry basis of the nanosuspension prepared with 1.5% w/v P188. The resultant powder was used alone or loaded into the eutectogels (Egel-PM).

### 2.9 Microscopical characterization

The morphologies of CUR, PM and CUR NCs were evaluated using a Tabletop TM 3030 scanning electron microscope (Hitachi, Tokyo, Japan).

### 2.10 Nanosuspensions stability

The physical stability of the NSs prepared with different P188 concentrations was assessed at room temperature and 4 ​°C. For this purpose, the NSs were kept in hermetically sealed, light-protected containers placed on the bench top or in the fridge, and samples were taken at times 0, 7, and 14 days. After a quick manual redispersion, 5 ​μL NS aliquots were taken, and the particle size, PDI, and zeta potential were measured using the technique described in the previous section. Results were expressed as mean values ​± ​standard deviation (mean ​± ​SD, *n* ​= ​3).

### 2.11 Thermal analysis

Differential scanning calorimetry (DSC) and thermogravimetric analysis (TGA) were performed on CUR, CUR NCs, and Egel-NCs. DSC runs were carried out in Q100 instrument (TA Instruments, New Castle, DE, USA) in a temperature range of 25–300 ​°C, with a heating ramp of 10 ​°C/min and a nitrogen flow of 10 ​mL/min. TGA experiments were carried out between 25 and 500 ​°C with a heating rate of 10 ​°C/min and a nitrogen flow of 10 ​mL/min, using a Q500 instrument (TA Instruments, New Castle, DE, USA).

### 2.12 Powder X-ray diffraction

X-ray diffraction was used to characterize the pure drug, the NCs and the NCs-loaded eutectogels in terms of crystalline state. For this purpose, a Miniflex® X-ray diffractometer was used (Rigaku Corporation, Kent, England), equipped with Cu Kα radiation. The scans were performed in a range of angles between 5° and 60° in 2θ in steps of 0.04, fixing the counting time at 0.5 ​s per step.

### 2.13 Probe tack test

Adhesion measurements were performed using a TA HD plus Texture Analyser equipment (Texture Technologies). Samples of 1.5 ​mm in thickness were fixed on a glass support. In the probe-tack tests, a circular Delrin® probe (10 ​mm Ø) comes into contact with the sample at a given velocity of 1 ​mm/s. A 500 ​g compressive force is applied for 1 ​s, and the probe is removed from the gel at a controlled velocity. For the eutectogels adhesion to mucin, a 10 ​mm Ø mucin disc was prepared by compressing the powder protein and attaching it to the probe tip using an ethyl-cyanoacrylate adhesive.

The debonding force and displacement were recorded and then converted to nominal stress and strain by normalizing the force by the probe area and the displacement by the initial thickness of the gel, respectively.

### 2.14 Eutectogel re-casting and NCs re-dispersion

The recycling ability of the eutectogels nanocomposites was evaluated by repeated cycles of heating above 50 ​°C/cooling at RT to induce their gel-sol phase transition. The gels were re-shaped in different forms by moulding the material at the liquid state. After each remoulding cycle, the redispersion of the CUR-NCs was assessed by dynamic light scattering (DLS).

### 2.15 3D printing of eutectogels

Egel-20 and Egel-20-NCs were printed into a mesh pattern using a 3D bio-printer (Bioscaffolder 3.2, GeSiM, Radeberg, Germany) equipped with a heated cartridge and a 0.25 ​mm nozzle. Both materials were placed into the cartridge and heated at 90 ​°C for 30 ​min before printing. Then, the temperature was allowed to equilibrate at 41 ​°C, and meshes were printed using a pressure of 300 ​kPa. The printing speed was 5 ​mm/s, and the layer height was 0.25 ​mm. The resultant meshes were observed in a digital microscope (VHX, Keyence, Ltd, Milton Keynes, UK) and a scanning electron microscope (Tabletop TM 3030, Hitachi, Tokyo, Japan). Furthermore, DSC experiments and redispersion of CUR-NCs from the meshes were performed to assess the effect of the 3D printing on the system.

### 2.16 In vitro drug release

The *in vitro* release profiles of coarse CUR, PM, CUR NCs and Egel-20-NCs were assessed using a dialysis membrane model according to a previously described methodology [27,28]. The release media consisted of 100 ​mL of 2% w/v Tween® 80 in buffer saliva pH 6.4 containing 0.5% w/v of ascorbic acid to prevent drug degradation [29]. For this experiment, 2.6 ​mg of CUR powder and CUR NCs (containing 1.97 ​mg of CUR) were placed in dialysis membrane bags of 12,000–14,000 ​kDa molecular weight cut-off (MWCO) (Spectra-Por, Spectrum Medical Industries, Los Angeles, CA, U.S.A.) and sealed at both ends with clips. Samples were placed in hermetically sealed Duran® bottles, which were placed in a ISF 7100 orbital incubator at 37 ​°C with an agitation speed of 100 ​rpm 400 ​μL-samples were withdrawn at chosen timepoints (5 ​h, 24 ​h, 4 days, 1 week, 2 weeks, 3 weeks, 4 weeks), replaced with fresh buffer, and quantified by high-performance liquid chromatography (HPLC). This experiment was performed in triplicate and the results expressed as means ​± ​SD. The drug release percentages were calculated using the following equation:

(1) $$\%\;of\;drug\;released=\frac{CUR\;quantified\;in\;the\;release\;media\;}{Initial\;amount\;of\;CUR}\times 100$$

### 2.17 Drug quantification by HPLC

CUR was quantified using reverse-phase HPLC on an Agilent instrument equipped with UV detection (Agilent Technologies 1220 Infinity UK Ltd, Stockport, UK). The column used was a ZORBAX Eclipse® XDB-C18 column (50 ​× ​4.6 ​mm internal diameter; 1.8 ​μm particle size) with a fixed temperature of 25 ​°C. The mobile phase comprised 80% acetonitrile and 20% phosphoric acid 0.1%. The maxima absorption (*λ*_max_) was fixed at 425 ​nm, the injection volume was 20 ​μL, and the flow rate was 0.5 ​mL/min. The linearity of the method was explored in the concentration range of 0.125–50 ​μg/mL (R^2^ ​= ​1), with a limit of detection of (LoD) of 0.40 ​μg/mL and a limit of quantification (LoQ) of 1.21 ​μg/mL.

### 2.18 Ex vivo mucosal penetration

As detailed below, the *ex vivo* mucosal deposition studies of Egel-20-NCs and Egel-20-PM were performed in excised buccal neonatal and adult porcine mucosa.

#### 2.18.1 Drug deposition in excised adult porcine mucosa

The excised mucosa was obtained from a local butchery shop, cleaned with pH 7.4 mucus buffer, and gently cut to the size of the Franz cells donor compartment with a sterile scalpel. Then, 6 ​mm eutectogels disks were placed on the mucosa, and the Franz cells' donor section was attached to each mucosa using cyanoacrylate glue. Subsequently, the system was mounted on the Franz cells and the receptor compartment was filled with 12 ​mL of 2% Tween® 80 in PBS with 0.5% ascorbic acid. Afterwards, 100 ​μL of buffer saliva was added into the donor compartment, which was closed using Parafilm® M to avoid water evaporation. The system's temperature was preserved at 37 ​± ​1 ​°C using a water circulator (Julabo Corio C, Cole Palmer, Vernon Hills, Illinois, USA). After 6 ​h, the system was disassembled, the excess formulation was gently removed, and the mucosa was rinsed with 1.5 ​mL pH 7.4 mucus buffer. Subsequently, an 8-mm biopsy punch was used to extract a section of the mucosa from where the drug was extracted. An illustration of the experimental setup for the mucosal deposition in excised neonatal mucosa is provided in Fig. S1A. This experiment was carried out in triplicate, and the results were expressed as means ​± ​SD.

#### 2.18.2 Drug deposition in excised buccal neonatal porcine mucosa

The mucosa was cleaned with pH 7.4 mucus buffer and gently cut with a sterile scalpel. Then, as detailed in previous publications, each mucosa section was placed in a weighing boat containing tissue paper soaked with mucus buffer [30]. Resin 3D printed rings with an inner diameter of 6 ​mm and 5 ​mm height were attached to the mucosa with cyanoacrylate glue using manual force for 30 ​s. Inside the ring, 6 ​mm disks of Egel-20-NCs or Egel-PM were added, followed by 10 ​μL of pH 6.4 saliva buffer. The system was sealed with Parafilm M® to avoid water evaporation and placed in an oven at 37 ​± ​1 ​°C. After 24 ​h, the rings and eutectogels were removed, the mucosa was rinsed with 1 ​mL pH 7.4 mucus buffer, and the excess formulation was wiped with a clean tissue paper wetted with mucus buffer. Subsequently, a 6 ​mm diameter biopsy punch was used to obtain a circular section of mucosa from where the drug was extracted. The manufacturing method for the ring insert is provided in supplementary method 1, and an illustration of the experimental setup for the mucosal deposition in excised neonatal mucosa is provided in Fig. S1B. This experiment was carried out in triplicate, and the results were expressed as means ​± ​SD.

#### 2.18.3 Drug extraction from mucosal tissues

Each circular section of mucosa was placed in a 2-mL Eppendorf tube with two stainless steel beads (0.5 ​cm diameter, Qiagen, Hilden, Germany) and 500 ​μL of purified water. Then the tubes were placed in a TissueLyser® LT (Qiagen, Hilden, Germany) and processed for 10 ​min at 50 ​Hz to hydrate the mucosa [27,31]. Afterwards, 1 ​mL of acetonitrile was added to each tube, and the process was repeated for another 10 ​min at 50 ​Hz to extract the drug. The homogenized tissue was centrifuged at 14,462 ​*g* for 10 ​min (Sigma microtube centrifuge SciQuip Ltd, Shropshire, UK), and the CUR content in the supernatant was analyzed by HPLC using the method detailed in Section 2.18.

#### 2.18.4 Fluorescent microscopy

Specimens of the mucosal tissues obtained from the drug deposition experiment in excised adult porcine mucosa were imaged in a multi-photon microscope (MPM) (Leica TCS SP8- multi-photon excited fluorescence upright microscope, Leica Micro-systems Ltd., Milton Keynes, UK) to observe the distribution of NR in the scleral tissue.

### 2.19 Statistical analysis

GraphPad Prism© software (version 8.0, GraphPad Software Inc, San Diego, California, USA) was used to build plots and perform statistical analyses. An unpaired *t*-test was applied when comparing two cohorts, whereas one-way ANOVA was applied to compare more than two cohorts. The results were expressed as means ​± ​SD, and in all cases, a *p*-value < 0.05 denoted significance.

## 3 Results and discussion

### 3.1 Preparation and characterization of gelatin-based eutectogels

**Fig. 2:**
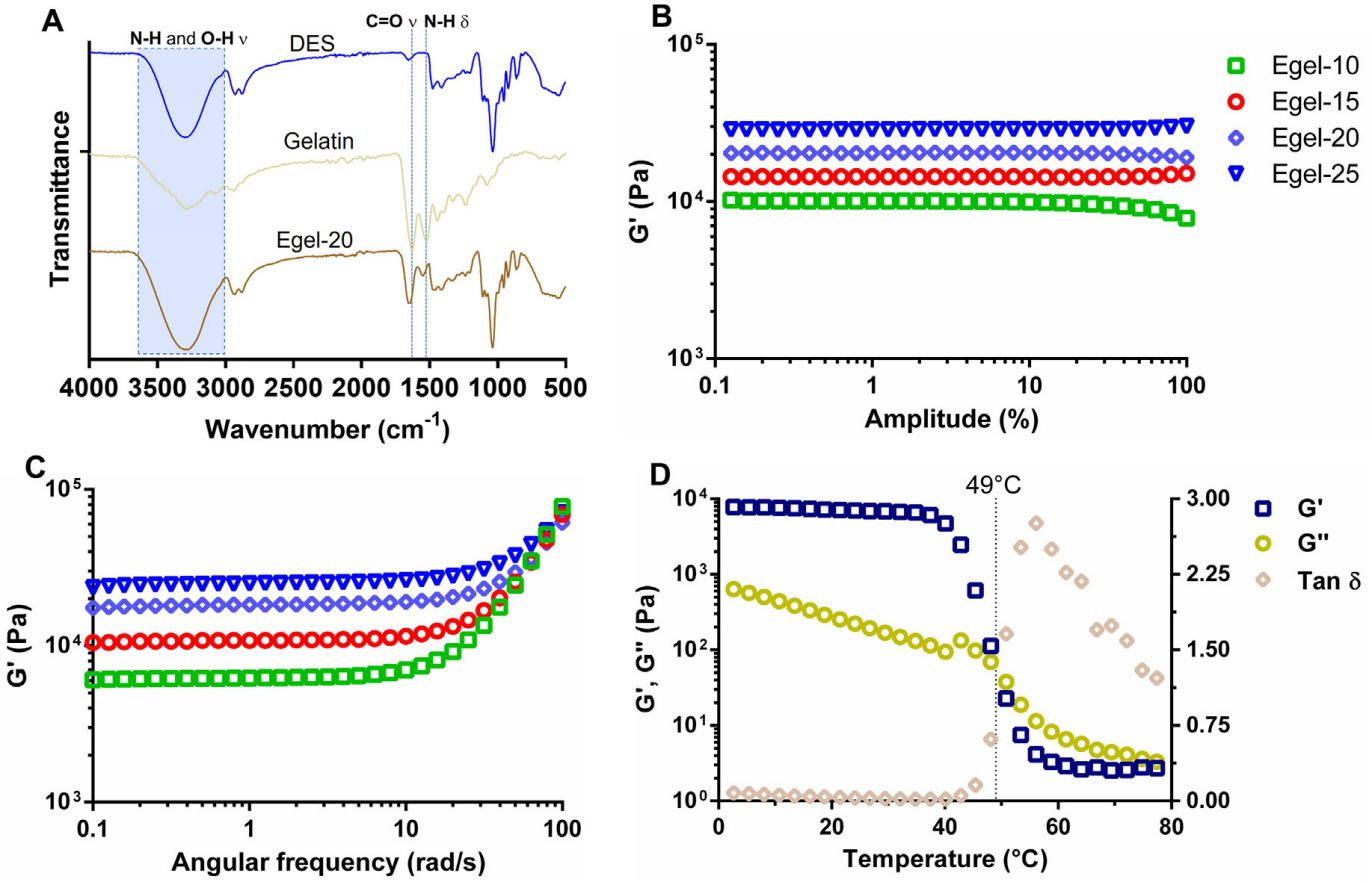
**A-** FTIR spectra of glycerine (ChCl/Gly 1:3), gelatin, and Egel-20 eutectogel. Amplitude **(B)** and frequency sweeps **(C)** for the as-prepared eutectogels. **D-** Evolution of the dynamic moduli and tan ​δ *vs* temperature for Egel-20.

The transition of gelatin chains from random coil to triple helix after heating/cooling steps is a well-known phenomenon inducing gelation. Recent studies have shown that gelatin can undergo this transition in some polyol-based DES, resulting in eutectogels with excellent stretchability [32,33]. Thus, we selected the eutectic mixture glycerine, ChCl: Gly (1:3), to drive the protein gelation. Gelatin and Gly have shown excellent compatibility, mainly promoted by polar interactions between –NH_2_ and –COOH groups from the protein and –OH groups from the polyol [5]. Indeed, FTIR analysis of Fig. 2A revealed a significant blue shift in the C=O stretching (from 1631 to 1650 ​cm^−1^) and *N*–H bending from 1525 to 1550 ​cm^−1^) bands of gelatin after eutectogel formation. Furthermore, a band broadening in the region 3000-3700 ​cm^−1^ corresponding to *N*–H and O–H stretching is also evidenced. All these changes in the vibrational modes indicate strong intermolecular interactions, including hydrogen bonding, between the eutectic mixture and the protein.

Obviously, the protein concentration can boost the formation of these non-covalent bonds and triple helixes, endowing the biopolymer gels with superior features. Therefore, we varied the gelatin concentration in the eutectogels from 10 to 25% w/v and explored the impact on the rheological and mechanical behaviour. As seen in Fig. 2B, amplitude sweeps show that the materials' elastic modulus (G′) increased from 10 to 29 ​kPa for Egel-10 and Egel-25, respectively. Besides, all the eutectogels showed a large linear viscoelastic range of over 100%. In the same line, frequency sweeps of Fig. 2C indicate that the eutectogel were sturdy materials in the range of 0.1–100 ​rad/s, featured by a marked increase in G′ at high frequencies. The stiffening of the eutectogel network can be attributed to free gelatin chains with long relaxation times, acting as physical crosslinkers of the gel matrix.

Due to the dynamic interactions between gelatin and Gly, the eutectogels benefit from a thermoreversible gel to sol phase transition, which endows these materials with remoulding and 3D-printing ability. As an example, the evolution of G′, viscous modulus (G″), and tan ​δ *vs* temperature for Egel-20 is shown in Fig. 2D. The gel-sol transition temperature (T_gel-sol_) was defined as the temperature at which G' ​= ​G''. An increasing trend of the T_gel-sol_ with the gelatin concentration was observed, which varied from 45 to 51 ​°C for Egel-10 and Egel-25, respectively (see Fig. S2 of the supplementary Information). With a T_gel-sol_ of ≈49 ​°C, Egel-20 was fully stable until 40 ​°C, the onset of G′, unveiling the suitability of this material to be used at the body temperature.

**Fig. 3:**
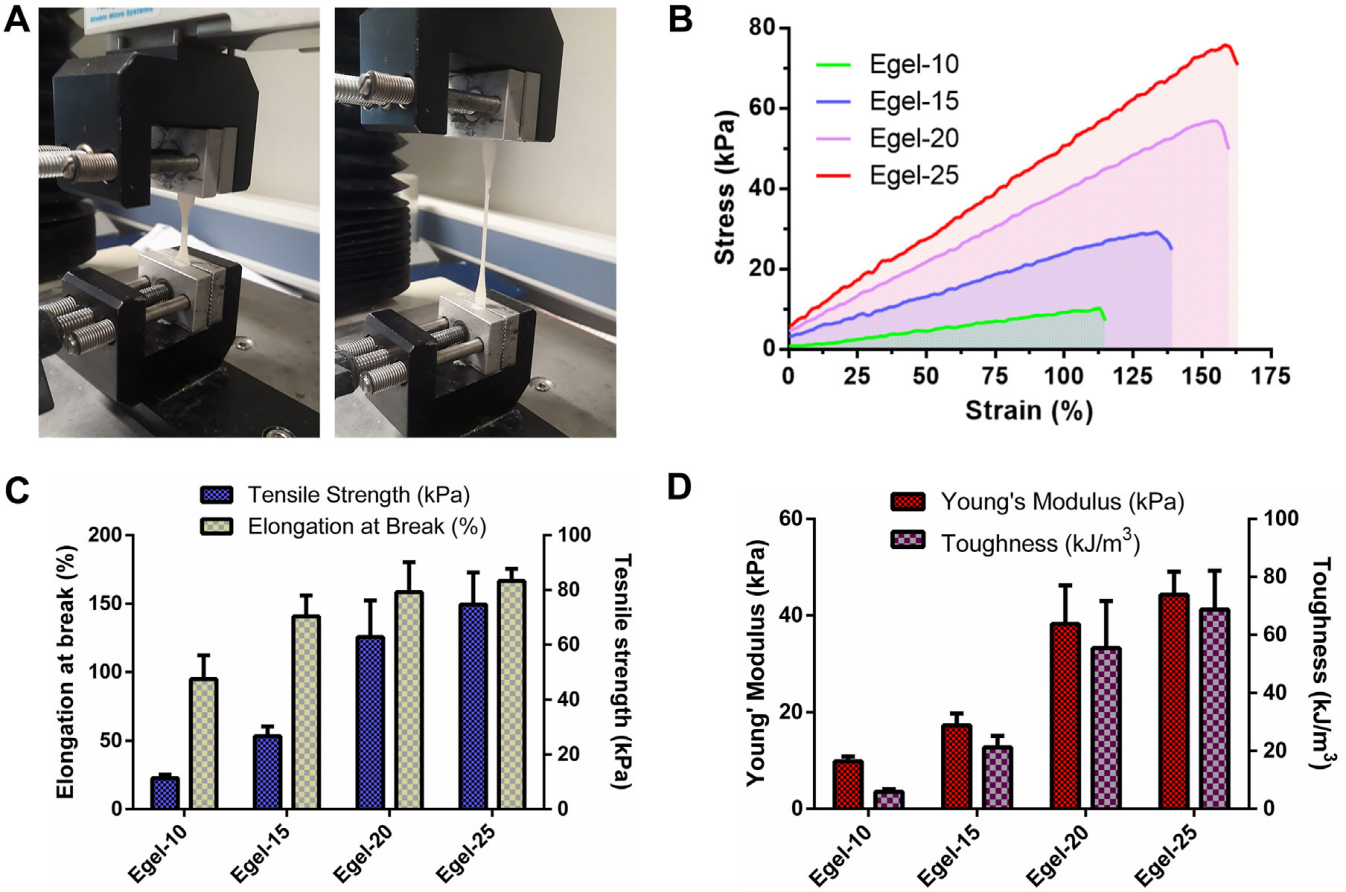
**A-** Photos of tensile test: eutectogel at rest (left) and during stretching (right). **B-** Stress *vs* strain curves for eutectogels with different gelatin concentrations. Elongation at break *vs* tensile strength **(C)** and Young's modulus *vs* toughness **(D)** for the as-prepared eutectogels. Results are expressed as means ​± ​SD for tensile strength, elongation at break, Young's modulus, and toughness (*n* ​= ​5).

Next, we investigated the mechanical properties of the eutectogels by tensile test, as shown in Fig. 3A. Fig. 3B presents the stress *vs* strain curves obtained for the eutectogels series, where an elastic behaviour was observed until the failure of the materials. In addition, an evident strengthening effect of the gelatin concentration can be observed, as the tensile strength and the elongation at break steadily increased from 11 ​kPa to 95% to 75 ​kPa and 167% for Egel-10 and Egel-20, respectively (Fig. 3C). Young's modulus also followed the same trend ranging from 10 to 45 ​kPa (Fig. 3D), which is in the range of the stiffness of biological tissues [34].

Due to the almost linear dependence between the protein concentration and the tensile strength and elongation at break, Egel-25 resulted in the toughest gel with a value of ≈70 ​kJ/m^3^ (Fig. 3D). However, the high viscosity of this formulation at the liquid state (above T_gel-sol_) could hinder the integration of drug NCs, turning the solution unhandy. Therefore, with a comparable toughness value of ≈55 ​kJ/m^3^, Egel-20 was selected as the most promising material to support the CUR NCs. The following sections will address the physicochemical properties of this drug nanocomposite eutectogel (Egel-20-NCs).

### 3.2 Manufacture of CUR-NCs

**Fig. 4:**
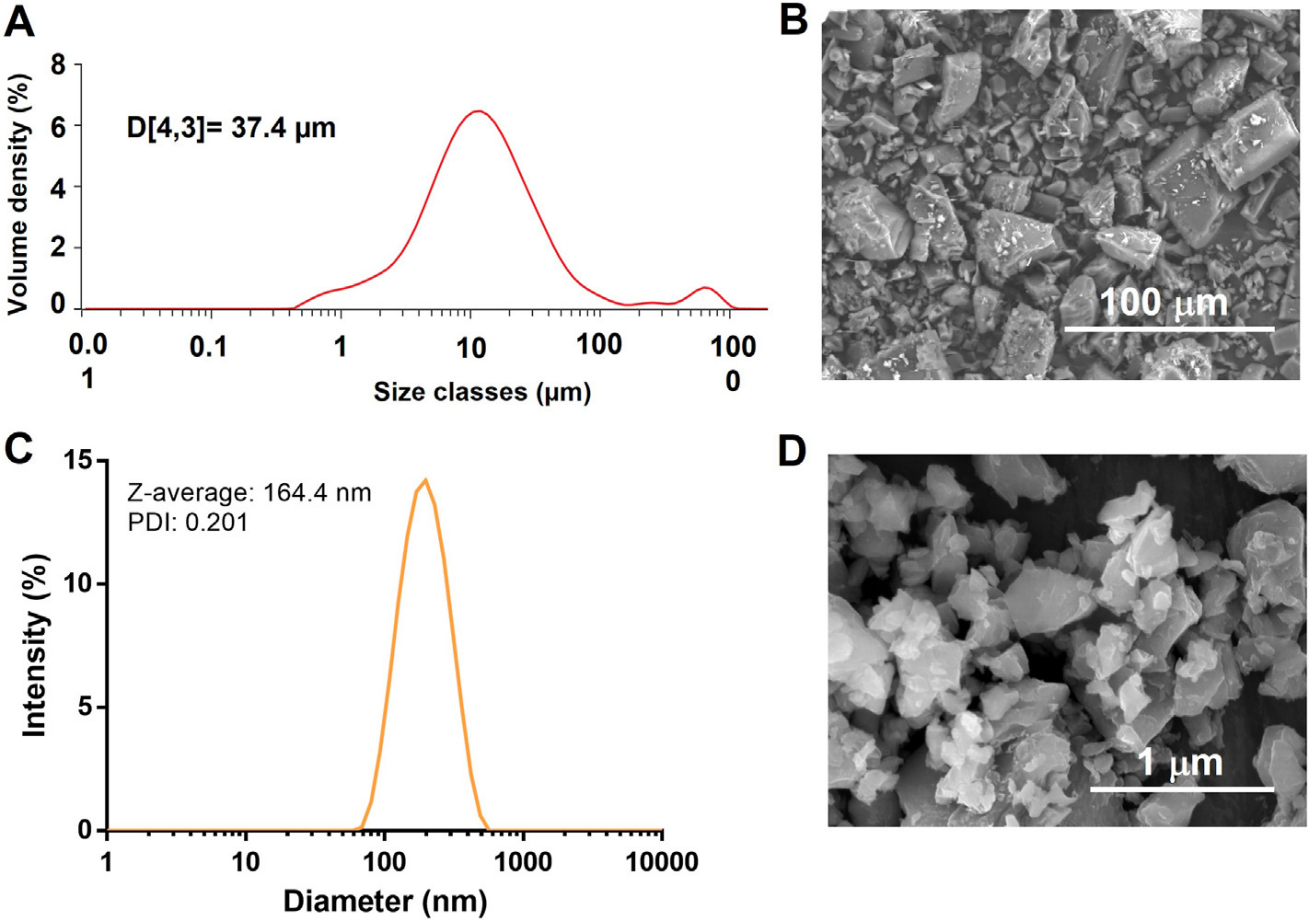
Production of CUR-NCs by media milling. **A**- Particle size distribution of coarse CUR obtained by laser diffraction; **B**- SEM images of coarse CUR. **C**- Intensity particle size distribution of CUR-NCs obtained by DLS. **D**- SEM images of CUR-NCs.

CUR NCs were successfully obtained using a simple lab-scale media milling technique. The particle size distribution of the coarse drug obtained by laser diffraction is presented in Fig. 4A, where a mean diameter D [3,4] of 37.4 ​μm and a bimodal volume distribution can be observed. This result was confirmed by SEM analysis of the coarse drug, as shown in Fig. 4B, where drug particles show heterogeneous sizes and cuboidal shapes. The milling process was efficient independently of the stabilizer concentration, since all the formulations presented particle sizes below 150 ​nm (0.5%w/v P188: 128.9 ​± ​11.9 ​nm, 1%w/v P188: 87.9 ​± ​3.0 ​nm, 1.5%w/v P188: 94.8 ​± ​3.1 ​nm, 2%w/v P188: 94.2 ​± ​6.5 ​nm). The PDI values were in all cases below 0.260, with no substantial differences regarding P188 concentration. Also, it is worth mentioning that the milling process did not degrade the drug's chemical composition, as demonstrated by FTIR analyses (Fig. S3 of the supplementary data).

Moreover, the zeta potentials were always negative, ranging from −12.8 ​± ​0.9 ​mV to −15.7 ​± ​2.2 ​mV. These results indicated that the concentration of the stabilizer did not play a critical role in the final properties of the formulations after 24 ​h of milling. Fig. 4C shows the particle size distribution of NCs containing 1.5% w/v of P188, where a monomodal distribution is observed with a Z-average diameter of 164.4 ​nm (PDI: 0.201). SEM images of this formulation evidenced uniform particles with sizes in good agreement with DLS results (Fig. 4D). The manufacture of NCs using laboratory media milling techniques has been previously described, allowing for the production of finely distributed particles in a scalable manner [35]. In this case, the particle size results agreed with those observed in previous reports using similar experimental setups [36]. During the milling process, there is an exponential increase in the surface area, which gives NCs some of their distinctive advantages, such as increased dissolution rate, saturation solubility and mucoadhesion [37]. However, NCs must remain stable to maintain all these advantages; hence, we studied the physical stability of the different NCs formulations.

### 3.3 Stability of CUR-NCs

**Fig. 5:**
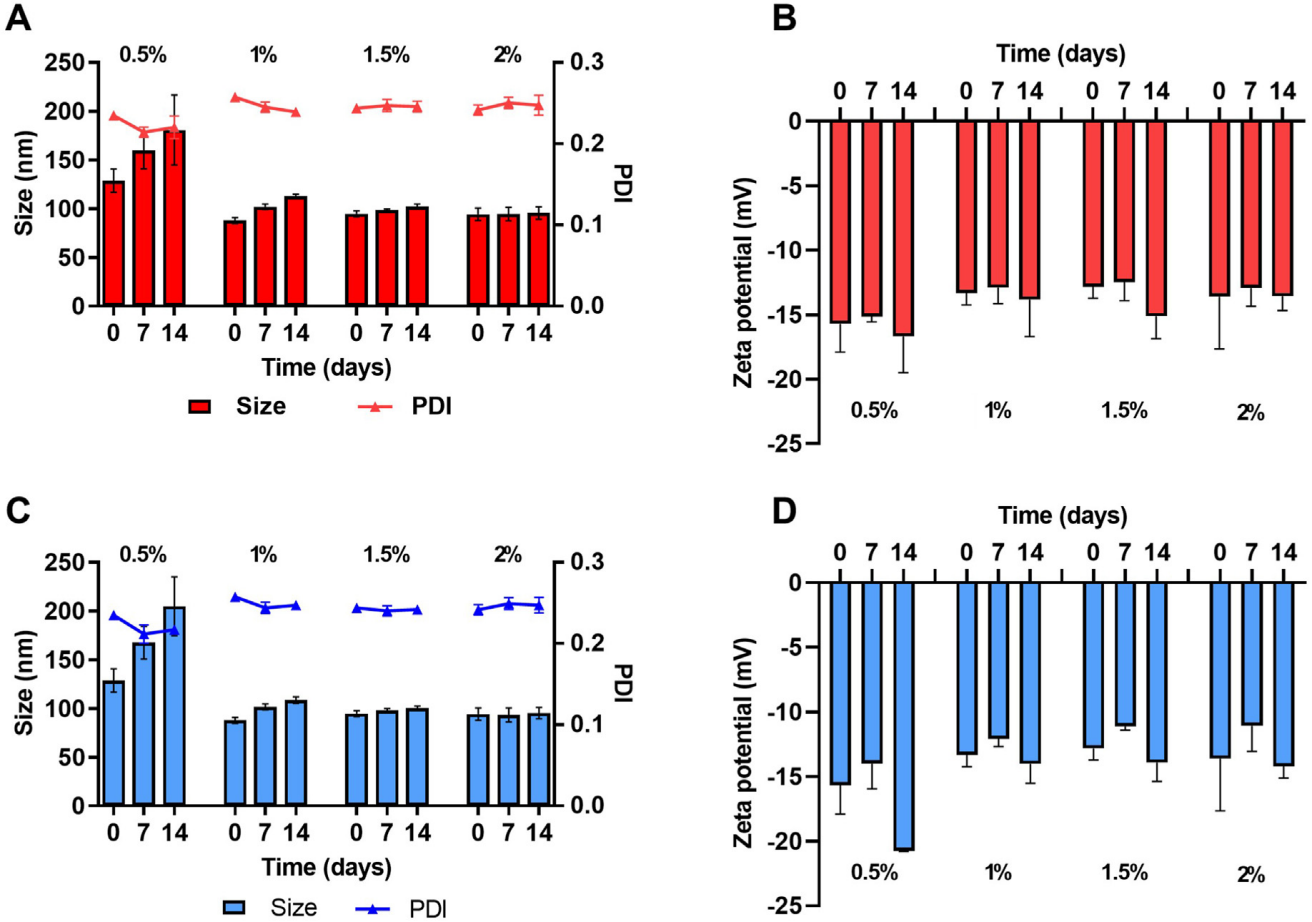
Stability of CUR-NCs stabilized with P188 0.5, 1, 1.5 and 2% w/v. **A**- Particle size and PDI of CUR-NCs at room temperature, **B**- Zeta potential of CUR-NCs at room temperature, **C**- Particle size and PDI of CUR-NCs at 4 ​°C, **D**- Zeta potential of CUR-NCs at 4 ​°C. Results are expressed as means ​± ​SD for PDI and means ​+ ​SD for particle size (*n* ​= ​3).

Fig. 5A and **C** shows the particle size of CUR NCs produced using increasing concentrations of P188 at room temperature and 4 ​°C, respectively. Both experiments showed similar trends, with a significant increase in particle size at a concentration of 0.5% w/v of P188, indicating the insufficient capacity of the system to keep the NCs stabilized over the evaluated period. On the contrary, increasing concentrations of the stabilizer led to improved stability, with negligible changes in the size, PDI and zeta potential over the evaluated period at concentrations of 1.5 and 2% w/v. Moreover, the zeta potential values remained negative for the duration of the study, in all cases with values greater than −10 mV, as shown in Fig. 5B and **D**. Ostwald ripening and particle aggregation can compromise the stability of the NCs and can be prevented by efficient stabilization of newly formed surfaces during the milling process [14,38]. Given the results observed in previous sections, the formulation containing 1.5% w/v of P188 was selected for further studies.

### 3.4 Physicochemical behaviour of the nanocomposite eutectogel

DES and NCs formulations have surprisingly improved the permeation and dissolution rate of class IV drugs. Herein, we hypothesized that these two technologies combined could act synergistically, rendering innovative pharmaceutical platforms with boosted delivery features. Besides, their application prospect is undoubtedly broadened by immobilizing the liquid and nanoparticulate formulations into an elastic and adhesive matrix.

**Fig. 6:**
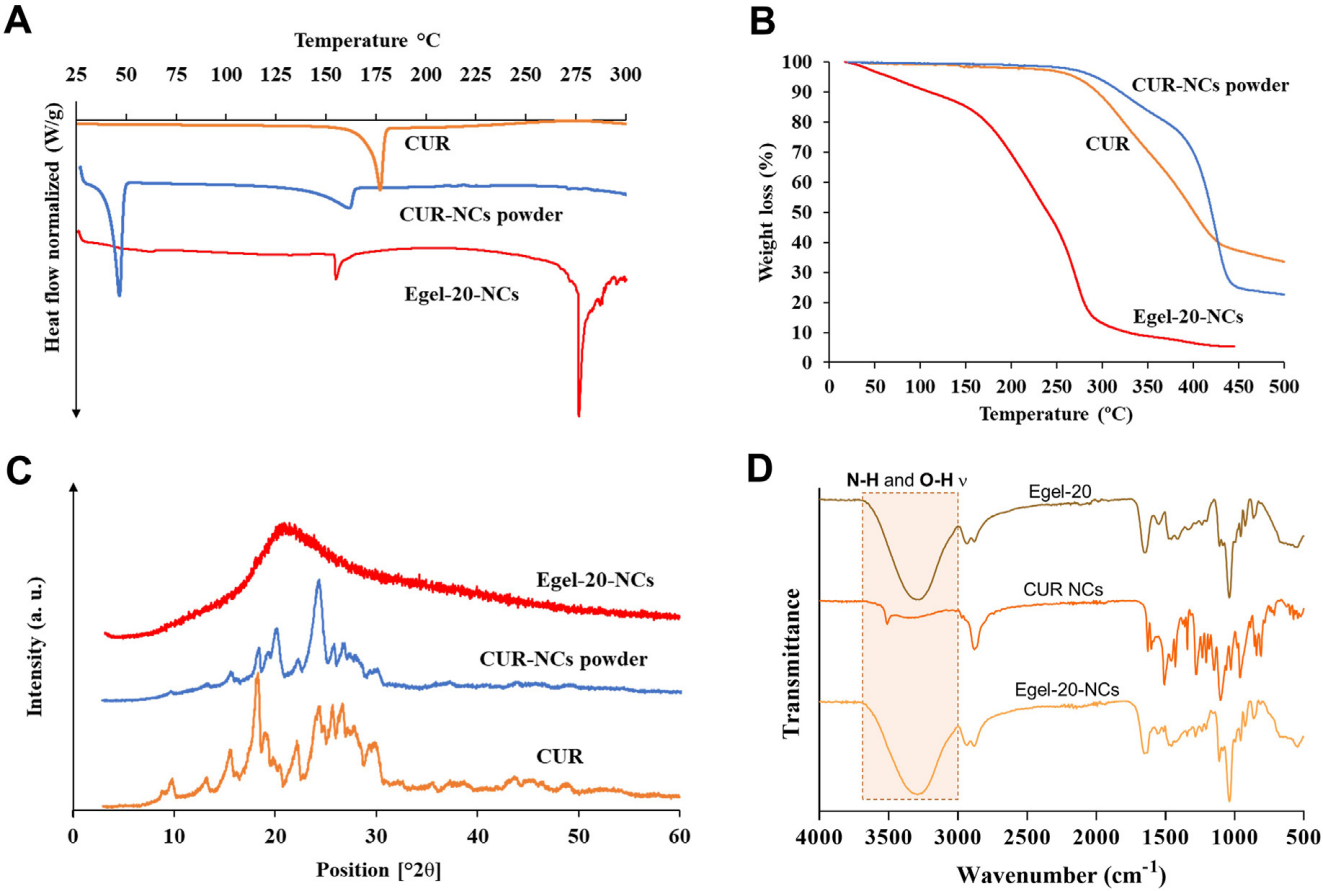
Physicochemical characterization of CUR, CUR-NCs, and Egel-20-NCs. **A**- DSC thermograms, **B**- Thermogravimetric traces, **C**- X-ray diffraction patterns, and **D**- FTIR profiles.

The study of the physicochemical characteristics of the drug and the formulation at different stages of the process is helpful to identify potential chemical instability, interactions with excipients, or changes in the crystalline state. Fig. 6A shows the DSC thermograms of CUR, CUR NCs and Egel-20-NCs. CUR is clearly crystalline as per the sharp endothermic peak observed at ≈175 ​°C, which corresponds with the melting point of the drug. After milling and freeze-drying, the NCs presented a melting peak at 52 ​°C, corresponding to P188. Moreover, the drug remained crystalline in the NCs, but the endothermic melting peak shifted to a lower temperature and decreased its intensity. This fact has been shown previously in similar formulations containing P188, where a partial thermally-induced amorphization occurred during the heating process in the DSC experiment, and P188 melts at the early stages of the experiment [14,39,40]. The NCs-loaded eutectogel also presented similar characteristics, a CUR melting peak at around 155 ​°C. This peak appears at similar temperatures than the one obtained for CUR NC formulations indicating that this molecule keeps a certain degree of crystallinity. Interestingly, the DSC curve shows an additional peak at around 275 ​°C, which could be due to the thermal degradation of the sample. Similar peaks have been reported for ChCl degradation in the past [41]. Further thermal characterization is presented in Fig. 6B, where the CUR and CUR NCs powders presented similar decomposition behaviour, starting around 300 ​°C. Egel-20-NCs, presented a gradual mass loss until 150 ​°C, probably associated with moisture evaporation, followed by a steeper mass loss associated with the decomposition of the system. This decomposition is consistent with the DSC peaks obtained at around 275 ​°C. Moreover, thermal degradation of gelatin has been reported to start between 250 and 300 ​°C. Interestingly, Gly degrades at higher temperatures [42]. Therefore, it can be established that the degradation observed in Fig. 6B can be attributed to ChCl and gelatin. The X-ray diffraction patterns (Fig. 6C) demonstrate that the pure CUR and the NCs form of the drug were clearly crystalline, with peaks observed across the evaluated range of 2θ degrees, i.e., at 12, 13, 18, and 24°. Expectedly, Egel-20-NCs showed the typical halo of amorphous materials, which is related to the relatively large proportion of amorphous excipients compared to CUR. Besides, the FTIR analysis shown in Fig. 6D demonstrated a good interfacial interaction between the drug and other components of the formulation. Characteristic peaks from the CUR NC formulation can be seen in the Egel-20-NCs FTIR spectrum, indicating the presence of the drug in the formulation. Finally, it is important to highlight that no signal shifts or new peaks were observed after gel formation, suggesting that the drug was not degraded during the formulation process.

**Fig. 7:**
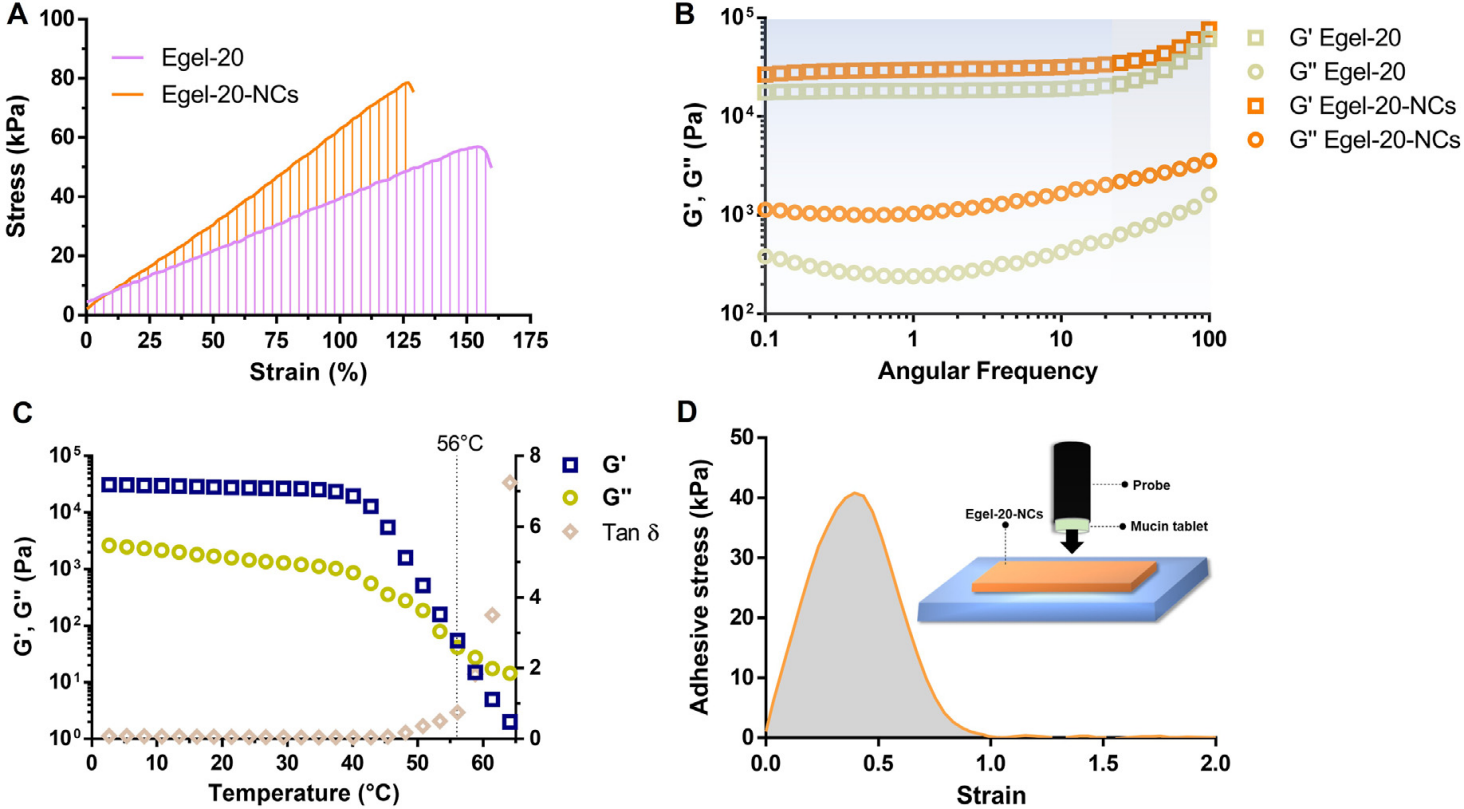
Stress *vs* strain curves **(A)** and frequency sweeps **(B)** for Egel-20 and Egel-20-NCs eutectogels. **C-** Evolution of dynamic moduli and tan d for the drug nanocomposite eutectogel. **D-** Adhesion curve of Egel-20-NCs on mucin protein as a substrate.

Interestingly, CUR NCs provided a reinforcement effect in the eutectogel matrix, increasing Young's modulus (38 *vs* 55 ​kPa) and tensile strength (63 *vs* 74 ​kPa) but decreasing its stretchability (158 *vs* 129 ​kPa) (Fig. 7A). Furthermore, frequency sweeps (Fig. 7B) showed that the incorporation of drug NCs also improved G′ in the rubbery plateau region (blue coloured) from ≈18 to 28 ​kPa. These results suggest that CUR NCs could act as physical crosslinking points, boosting the eutectogel network's stability. Indeed, the temperature sweeps in Fig. 7C revealed an increase in the T_gel-sol_ up to 56 ​°C, expanding the applicability range of temperature of the eutectogel nanocomposite.

Since the great number of polar groups in gelatin and glycerol (-COOH, –NH_2_, –OH, etc.), the eutectogel nanocomposite resulted in a highly adhesive material, as shown in Video S1 of the Supplementary data. Bioadhesiveness is a key-sought specification for mucus drug delivery, and these eutectogels could be attractive therapeutic platforms for this application. Next, we explored the adhesion properties of Egel-20-NCs to mucin protein, finding an excellent adhesive strength of ≈40 ​± ​7 ​kPa (Fig. 7D), outperforming other recently reported mucoadhesive hydrogels [[43], [44], [45]].

Supplementary data related to this article can be found at https://doi.org/10.1016/j.mtbio.2022.100471.

The following is the supplementary data related to this article:

Multimedia component 2 Multimedia component 2

### 3.5 Eutectogel-NCs remoulding and NCs re-dispersion

**Fig. 8:**
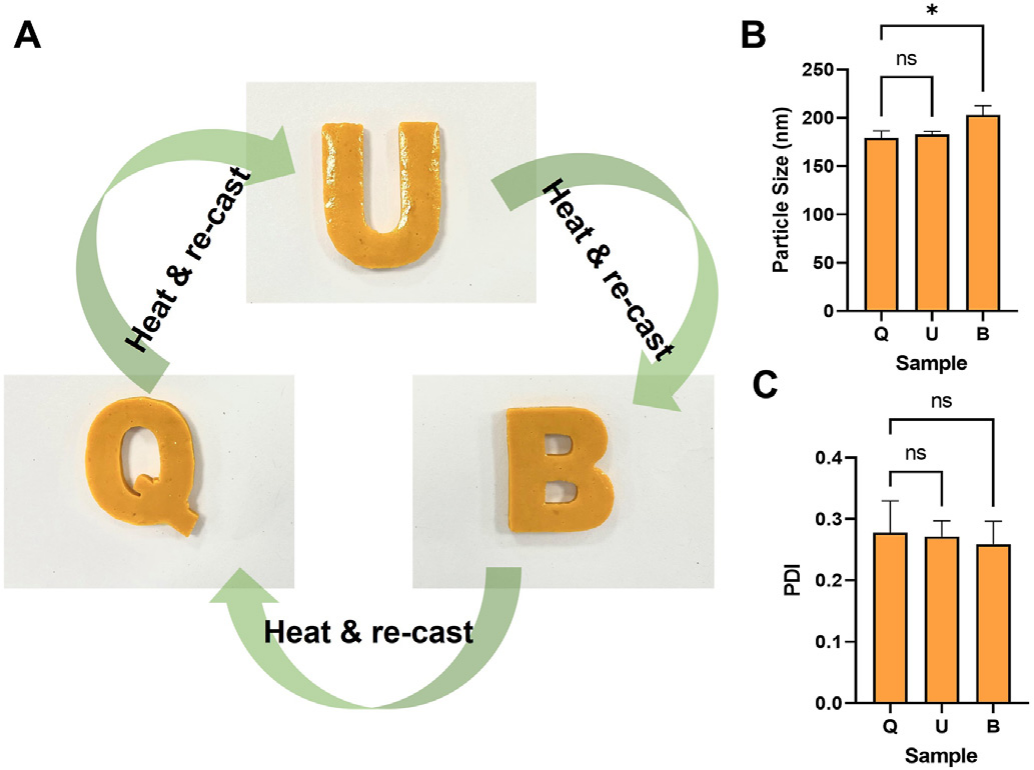
Egel-20-NCs casting multiple times and NCs re-dispersion. Multiple casting of Egel-20-NCs into moulds with Q-U-B shapes. **B**- Mean particle size of CUR-NCs after redispersion of Egel-20-NCs cast three times, **C**- PDI of CUR-NCs after redispersion of Egel-20-NCs cast three times. Data expressed as means ​± ​SD (*n* ​= ​3).

One of the most remarkable characteristics of these gelatin-based eutectogels is their ability to liquefy above the T_gel-sol_ and re-cast into a gel form multiple times. We evaluated the capacity of the NCs to redisperse from remoulded Egel-20-NCs. The casting and re-casting of the nanocomposite eutectogel using the “Q-U-B″ moulds led to apparently uniform systems with well-defined edges and shapes, as shown in Fig. 8A. The particle size of the NCs redispersed from the first, second and third casting (corresponding to the Q, U, and B shapes, respectively) is presented in Fig. 8B. The data indicate that no significant differences were observed in the particle size of the CUR between the first and second casting, whereas a slightly larger NCs size was observed for the third casting. Although the difference in this last case was significant (*p* ​= ​0.842), the mean particle remained between 187 and 213 ​nm. A similar trend was observed for the PDI of the NCs after redispersion (Fig. 8C), with values between 0.208 and 0.307, and no significant differences were observed between the analyzed samples (*p* ​< ​0.05 in both cases). This data indicates that the NCs would be readily available for dissolution and absorption upon contact with biological fluids, even after thermal manipulation of the gel during the manufacturing process.

### 3.6 3D printing of eutectogels

**Fig. 9:**
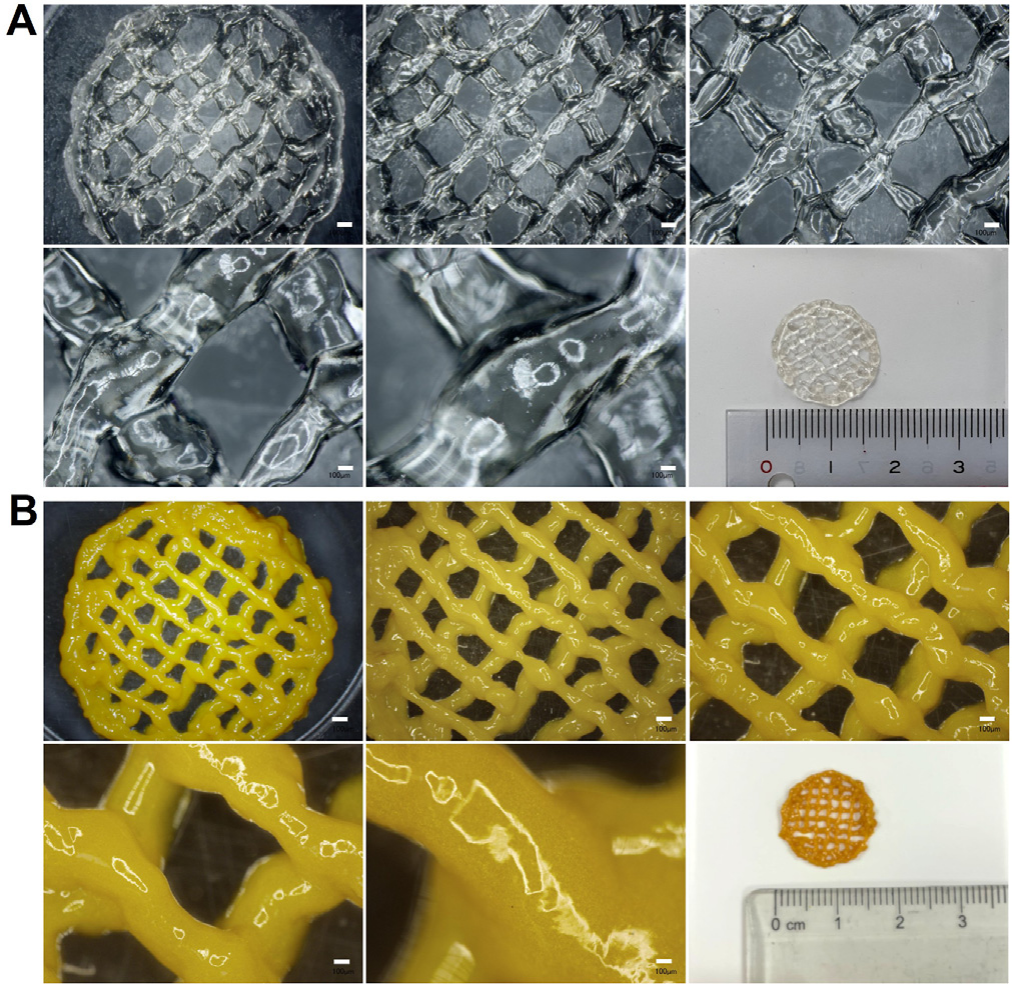
Optical microscopy images of 3D printed eutectogel meshes. **A-** Meshes obtained with blank eutectogels, **B-** Meshes obtained with Egel-20-NCs. Scale bar in all cases: 100 ​μm.

Additive manufacturing is a powerful technique for reducing the time, material waste, and energy needed to fabricate medical devices and is being actively applied for personalized biomedicine [46,47]. The good stability of CUR-NCs after several remoulding steps for Egel-20-NCs opens the gate for exploring the potential of these versatile materials in 3D-printing applications. Blank eutectogels and Egel-20-NCs were patterned into two-layered circular meshes with external diameters of 17 ​mm. Optical microscopy images of the blank eutectogels and Egel-20-NCs can be observed in Fig. 9A and B, respectively. In both cases, the gels flowed sufficiently well through the bioprinter's nozzle to form the designed pattern. Optical microscopy images revealed that the CUR-NCs were well distributed in the blend since the colour was homogeneous across the sample. Crucially, the thickness of both materials allowed the addition of a second layer on top of a first printed layer, highlighting the potential for constructing three-dimensional objects based on eutectogels. This result agrees with a recent report, where a semiconducting eutectogel was 3D printed as a wearable sensor [5].

**Fig. 10:**
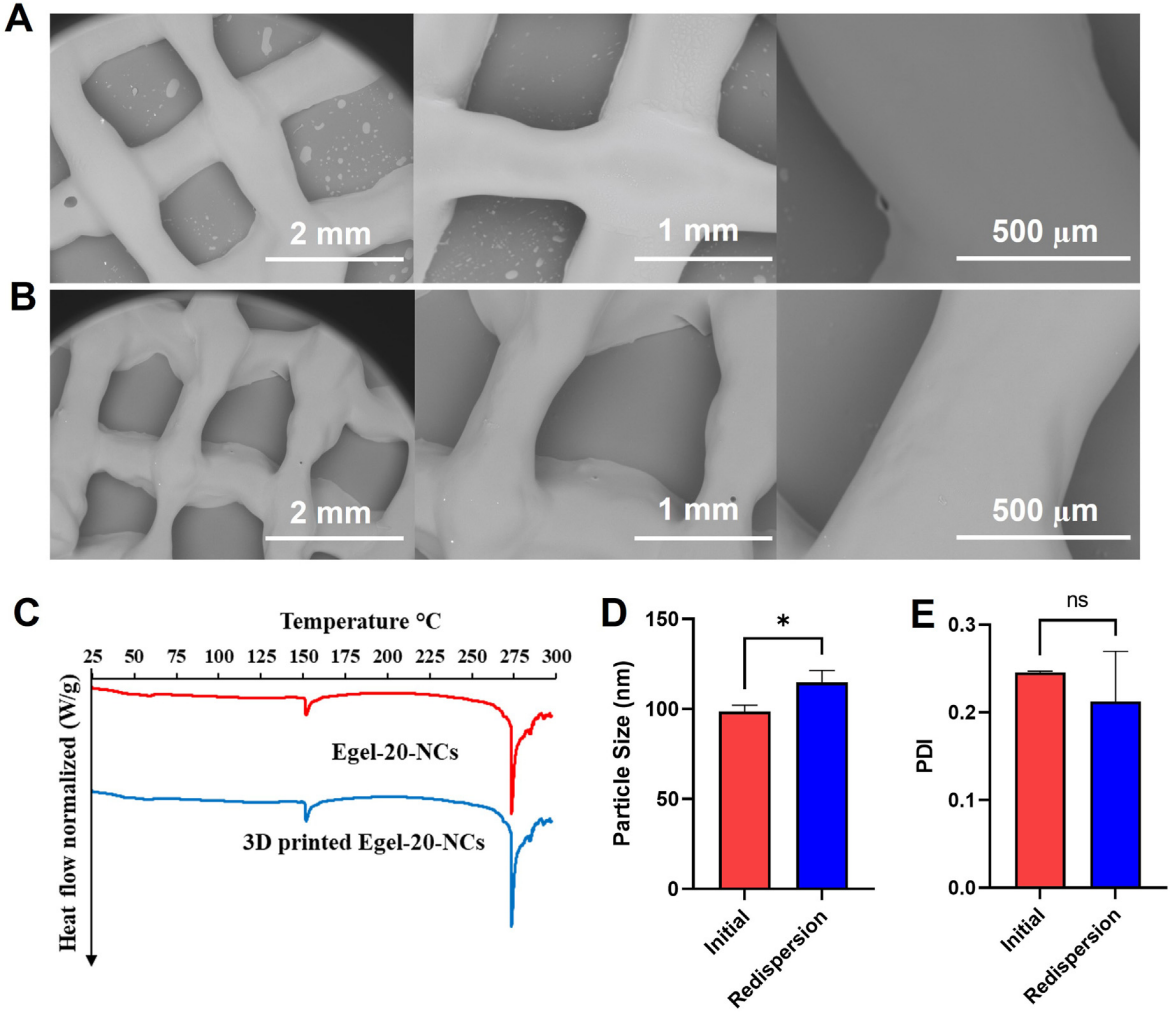
Physicochemical characterization of blank eutectogels and Egel-20-NCs. **A**- SEM images of blank eutectogels. **B**- SEM images of Egel-20-NCs. **C**- DSC traces of CUR-NCs and 3D printed Egel-20-NCs. **D**- Particle size of CUR-NCs and redispersed NCs from 3D printed Egel-20-NCs. **E**- PDI of CUR-NCs and redispersed NCs from 3D printed Egel-20-NCs. Data expressed as means ​± ​SD (*n* ​= ​3).

SEM was used to assess the morphology of the 3D printed meshes made of blank eutectogels and Egel-20-NCs, and the images are presented in Fig. 10A and **B**, respectively. Both formulations presented smooth surfaces with the absence of bubbles or particle aggregates. Moreover, the CUR NCs were not detectable in these images at the used magnifications. The SEM images also showed that the meshes were clearly formed, with uniform angles and strands.

Shear stress and temperature applied during the printing process could affect the printed materials' stability, which makes assessing their physicochemical properties critical. Fig. 10C shows the DSC traces of the Egel-20-NCs before and after printing, where the characteristic endothermic peaks were present in the evaluated range of temperatures. Fig. 10D and **E** shows the redispersion of the NCs from the 3D printed gels in terms of mean particle size and PDI, respectively. Although significant differences were observed between samples in terms of particle size, the redispersed NCs were only 16 ​nm bigger than those from the original samples. Similar behaviour is consistent with the particle sizes obtained after several cycles of melting and casting (section 3.5). Therefore, it is not strange to obtain a slightly larger particle size after heating the samples prior to 3D printing. Moreover, no significant differences were observed between the original and redispersed samples in terms of PDI.

### *3.7* *In vitro* drug release and mucosal deposition

**Fig. 11:**
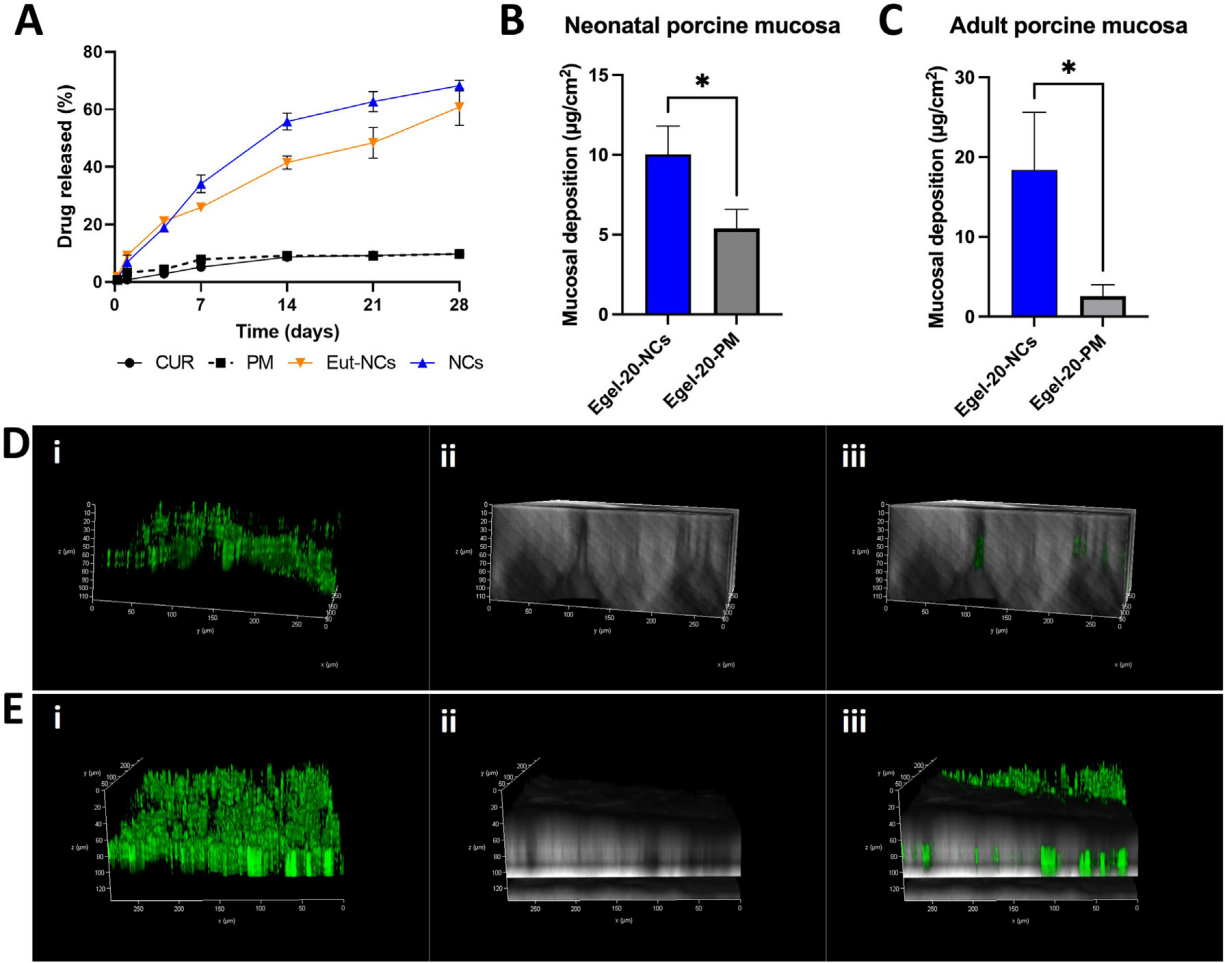
Drug release and mucosal deposition of Egel-20-NCs and Egel-20-PM. **A**- *In vitro* CUR release from CUR, CUR-NCs, Egel-20-NCs, and Egel-20-PM using dialysis membrane models. Results are expressed as means ​± ​SD (*n* ​= ​3). **B**- CUR deposition using Egel-20-NCs and Egel-20-PM in neonatal porcine mucosa, results expressed as means ​± ​SD (*n* ​= ​3). **C**- CUR deposition using Egel-20-NCs and Egel-20-PM in adult porcine mucosa, results expressed as means ​± ​SD (*n* ​= ​3). **D**- Multi-photon microscopy of neonatal porcine mucosa treated with Egel-20-PM, including (**i**) green fluorescent channel, (**ii**) transmitted light channel, and (**iii**) merged channels. **E**- Multi-photon microscopy of neonatal porcine mucosa treated with Egel-20-NCs, including (**i**) green, fluorescent channel, (**ii**) transmitted light channel, and (**iii**) merged channels.

The release profile of CUR from the pure drug, drug plus P188 physical mixture (PM), CUR NCs, and Egel-20-NCs is presented in Fig. 11A. The pure drug and PM presented very similar release profiles, reaching a maximum of only ≈10% in 28 days, which indicates the dissolution rate enhancement related to the stabilizer is negligible. Importantly, the incorporation of CUR in the PM did not produce any significant effect on its physicochemical properties, as revealed by the FTIR and particle size analyses presented in Fig. S3, and Fig. S4, respectively. CUR NCs and Egel-20-NCs presented substantially higher drug release rates, with 55.8 ​± ​2.9% and 41.5 ​± ​2.3% at day 14, respectively. By the end of the study, the amount of drug dissolved from the NCs-based formulations was not significantly different, achieving a maximum of 68.2 ​± ​1.9% and 60.9 ​± ​6.5% for NCs and the eutectogel formulation, respectively. Critically, the Egel-NCs formulation did not outperform the NCs alone, which can be possibly attributed to the presence of the Egel matrix, through which the drug once dissolved must diffuse in order to be released. CUR is a highly hydrophobic drug, and low release rates have been observed previously in similar experiments [48]. However, the formulation of NCs leads to an exponential increase in the specific surface and, thus, an increase in the dissolution rate according to the Noyes-Whitney equation [49]. Interestingly, these results prove the ability of the *N*C-based formulations for long-acting drug release.

The mucosal deposition in neonatal porcine mucosa is presented in Fig. 11B, where the Egel-20-NCs was able to deposit twice the amount of drug that was deposited by a eutectogel formulated with the PM, again highlighting the positive effect of particle size reduction, especially regarding the enhanced adhesion to biological surfaces [50]. A similar trend was observed in adult porcine mucosa, where the Egel-NCs deposited an amount of CUR 8-fold higher than that for Egel-PM (Fig. 11C). The differences in the magnitude of drug deposition between the two mucosal models could have been related to the integrity of the tissue and composition of the buccal fluids. However, the objective of this experiment was to explore methods for assessing mucosal drug deposition using novel eutectogels.

The observation of the adult mucosa by multimodal multi-photon imaging (MPM) further confirmed the increased drug deposition for Egel-20-NCs (Fig. 11D) in comparison with the Egel-PM (Fig. 11E). In this experiment, the green fluorescence observed in the microscopical images corresponds to the CUR autofluorescence. Figures D and E (i) correspond to the drug fluorescence only, D and E (ii) to the mucosa only, and D and E (iii) to both channels merged. Furthermore, the three-dimensional photomicrographs revealed that the NCs-based formulation enabled penetration of the drug to deeper layers in the mucosa, with fluorescent traces observed up to 100 ​μm in depth, whereas the Egel-PM led to lower CUR penetration (∼60 ​μm). Altogether, these results prove that this new platform could be used to deliver hydrophobic drugs for prolonged periods to mucosal tissues and that the reduction in the particle size was critical for increasing drug deposition.

The unique properties of these materials could allow a wide range of applications across drug delivery beyond mucosal tissues. One interesting example is drug delivery to wounds, which could be achieved in an effective manner given the biocompatibility, bioadhesion, and low water content of these gels, with the consequent reduced risk of microorganism contamination. In particular, the antioxidant, anti-inflammatory, and antibacterial properties of CUR would be attractive for this application [51]. Moreover, these systems have low volatility compared to regular hydrogels that eventually dry and make it difficult the removal from the wound area [52]. Finally, 3D printing would enable the preparation of patches or meshes to treat wounds in a personalized fashion.

## 4 Conclusion

A new drug delivery platform was developed in this work, combining emerging technologies of DES and drug NCs. We demonstrated that the viscoelastic and mechanical properties of gelatin/glycerine eutectogels could be tailored by modulating the gelatin concentration in the formulation. Interestingly, eutectogels integrating curcumin NCs showed excellent elasticity and stretchability, and they benefited from low reversible gel-sol transition temperature (56 ​°C). This attractive feature enabled the manufacture of 3D printed meshes while allowing the complete redispersion of the NCs from the gels. The drug nanocomposite eutectogel showed excellent adhesiveness to mucin, impelling a long-acting drug release profile and an enhanced capacity to deliver the hydrophobic CUR to porcine mucosa. Interestingly, coarse CUR supported in the eutectogel showed considerably lower mucosal deposition than the equivalent CUR NCs formulation, revealing the positive effect of combining both DES and nanodispersion technologies.

All in all, these novel therapeutic nanocomposite eutectogels open new directions in the rational design of innovative drug delivery systems, where drug NCs and an assortment of DES could be synergistically combined and applied to manage different diseases. Notably, future research should evaluate the performance of these materials in a specific disorder developing in mucus environments like bacterial sinusitis, bacterial vaginosis, or mouth sores.

## Credit author statement

**María Beatrice Bianchi**: Methodology, Investigation, Formal analysis, Writing-Original draft. **Chunyang Zhang:** Methodology, Investigation. **Elise Catlin**: Methodology, Investigation. **Giuseppina Sandri:** Writing - review & editing. **Marcelo Calderón:** Writing - review & editing. **Eneko Larrañeta:** Methodology, Investigation, Writing - review & editing. **Ryan F. Donnelly**: Writing - review & editing. **Matías L. Picchio**: Methodology, Investigation, Writing-Original draft, Writing - review & editing. **Alejandro J. Paredes**: Methodology, Investigation, Conceptualization, Supervision, Funding acquisition, Writing-Original draft, Writing - review & editing.

## Declaration of competing interest

The authors declare that they have no known competing financial interests or personal relationships that could have appeared to influence the work reported in this paper.

## Data Availability

Data will be made available on request.
