# Supplementary material for: Bioadhesive eutectogels supporting drug nanocrystals for long-acting delivery to mucosal tissues
Source: Mater Today Bio. 2022 Oct 25;17:100471. doi: 10.1016/j.mtbio.2022.100471 (PMC9636571; doi:10.1016/j.mtbio.2022.100471)
Supplement: Multimedia component 1 [file mmc1.docx]

**Supplementary Material**

**Bioadhesive eutectogels supporting drug nanocrystals for long-acting delivery to mucosal tissues**

María Beatrice Bianchi^1^, Chunyang Zhang^2^, Elise Catlin^2^, Giuseppina Sandri^1^, Marcelo Calderón^3,4^, Eneko Larrañeta^1^, Ryan F. Donnelly^1^, Matías L. Picchio^3*^, Alejandro J. Paredes^2^*

^1^ Department of Drug Sciences, University of Pavia, Viale Taramelli 12, 27100 Pavia, Italy.

^2^ School of Pharmacy, Queen's University Belfast, Medical Biology Centre, 97 Lisburn Road, Belfast. BT9 7BL, UK.

^3^ POLYMAT, Applied Chemistry Department, Faculty of Chemistry, University of the Basque Country UPV/EHU, Paseo Manuel de Lardizabal 3, 20018 Donostia-San Sebastián, Spain.

^4^ IKERBASQUE, Basque Foundation for Science, 48009 Bilbao, Spain

***Corresponding authors:**

Dr. Matías Picchio

POLYMAT Institute - University of the Basque Country UPV/EHU

Responsive Polymer Therapeutic Group

Faculty of Chemistry

Paseo Manuel de Lardizabal 3, 20018

Donostia-San Sebastián · Spain

Phone: +34 943 01 53 25

Email: [matias.picchio@polymat.eu](mailto:matias.picchio@polymat.eu)

Dr. Alejandro J. Paredes

Lecturer in Pharmaceutical Sciences

School of Pharmacy

Queen's University Belfast

Medical Biology Centre

97 Lisburn Road

Belfast, BT9 7BL

United Kingdom

Tel: +44(0)2890971061

Email: [a.paredes@qub.ac.uk](mailto:a.paredes@qub.ac.uk)

**Supplementary methods**

**SM1. Fabrication of ring inserts for mucosal drug deposition**

The holding rings were produced based on 3D printing technology. Prior to printing, the dimension of the ring was designed using thinker CAD software. The ring was designed with an inner diameter of 8 mm, outer diameter of 12 mm, and the height of 7 mm. The resin 3D printer (Anycubic Photon S) was applied to print the rings, using Anycubic UV sensitive resin (405 nm). Afterwards, the printed rings were removed from the printing plate and cured using UV light (405 nm) for 15 min.

**SM2. Preparation of buffers**

Two different types of buffers were prepared to run the in vitro release and ex vivo tests. The first buffer was used to simulate the mucus from mucosal tissues in the mouth, and it consisted of a pH 7.4 saline isotonic solution (KH_2_PO_4_ 1.90 g/l; Na_2_HPO_4_ 8.10 g/l; NaCl 4.11 g/l). The second prepared was a pH 6.4 phosphate buffer (USP 25), used to simulate the saliva and the buccal environment.

**Supplementary Figures**


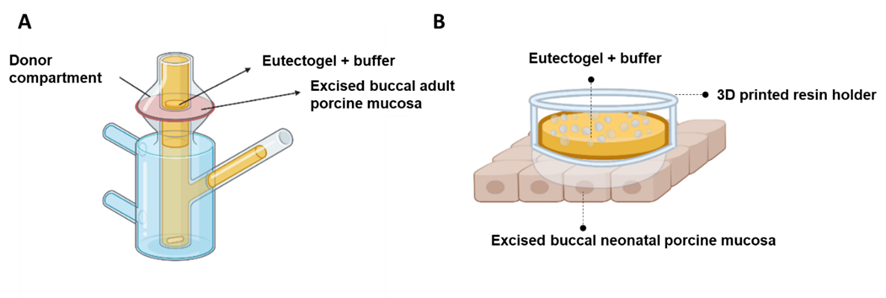
**Figure S1**. Drug deposition in porcine mucosa. A- Drug deposition in excised buccal adult porcine mucosa, and B- Drug deposition in excised neonatal porcine mucosa.


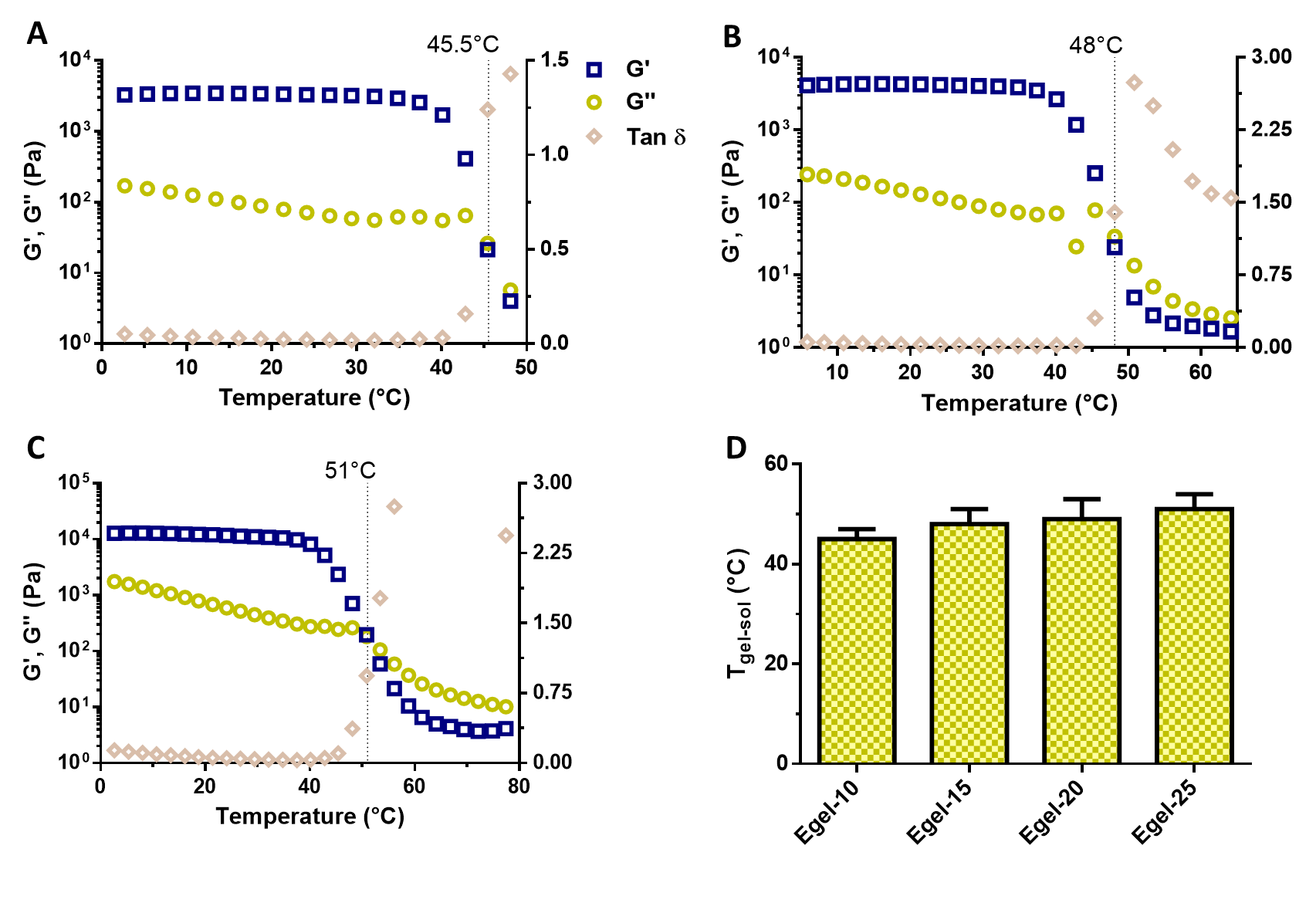


**Figure S2.** Temperature sweeps for Egel-10 (**A**), Egel-15 (**B**), and Egel-25 (**C**). **D-** Gel to sol phase transition temperatures for the as-prepared gelatin-based eutectogels.

**Figure S3.** FTIR spectra of CUR, CUR NCs, and PM.

Note that the signal at around 2890 cm^-1^ in CUR NCs corresponds to the surfactant P188 used for stabilizing the nanodispersion (P188). All the characteristic peaks of the drug are present in the NCs and PM, indicating no drug degradation or chemical interaction with excipients.


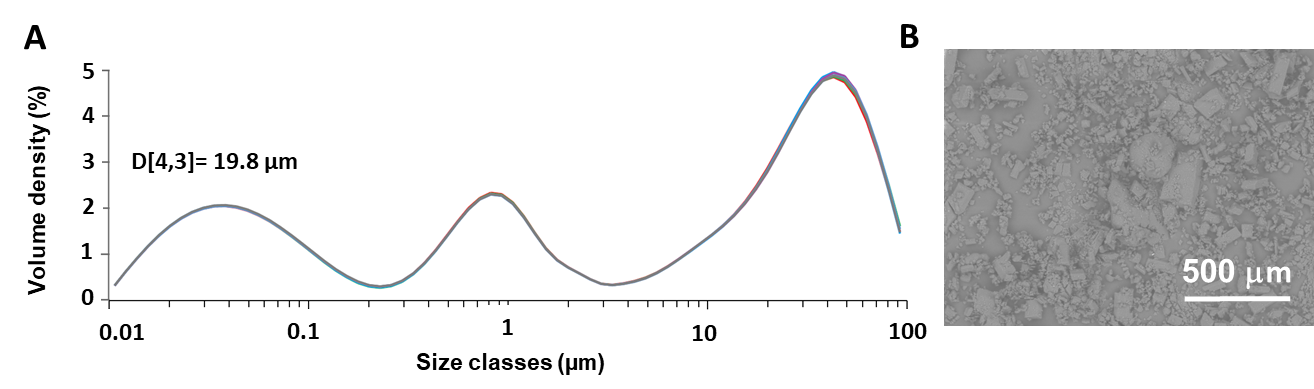
 **Figure S4**. A- Particle size distribution of the physical mixture (PM) composed of CUR and P188, obtained by laser diffraction, results expressed as mean ± S.D., (*n* = 6). B- Scanning electron microscopy photomicrographs of the same physical mixture.

Moreover, the mean particle size of the drug in the PM, evaluated in aqueous dispersion by laser diffraction, was 19.8 µm (D[4,3]). The slight decrease in the mean particle size when compared to the pure drug is related to a higher concentration of P188 during measurement, enabling a better particle de-aggregation and dispersion. SEM images revealed large aggregates made of P188 granules surrounded by CUR particles (approximately 200 µm in size and spherical shape).

**Supplementary video 1 is provided in the online submission**
